# Immunosuppression Affects Neutrophil Functions: Does Calcineurin-NFAT Signaling Matter?

**DOI:** 10.3389/fimmu.2021.770515

**Published:** 2021-11-02

**Authors:** Ondřej Vymazal, Kamila Bendíčková, Marco De Zuani, Marcela Vlková, Marcela Hortová-Kohoutková, Jan Frič

**Affiliations:** ^1^International Clinical Research Center, St. Anne’s University Hospital, Brno, Czechia; ^2^Department of Biology, Faculty of Medicine, Masaryk University, Brno, Czechia; ^3^Department of Clinical Immunology and Allergology, Faculty of Medicine, Masaryk University, Brno, Czechia; ^4^Department of Clinical Immunology and Allergology, St. Anne´s University Hospital, Brno, Czechia; ^5^Department of Modern Immunotherapy, Institute of Hematology and Blood Transfusion, Prague, Czechia

**Keywords:** immunosuppression, calcineurin inbibitors, neutrophil (PMN) function, sepsis, NFAT signaling, pattern recognition receptor (PRR)

## Abstract

Neutrophils are innate immune cells with important roles in antimicrobial defense. However, impaired or dysregulated neutrophil function can result in host tissue damage, loss of homeostasis, hyperinflammation or pathological immunosuppression. A central link between neutrophil activation and immune outcomes is emerging to be the calcineurin-nuclear factor of activated T cells (NFAT) signaling pathway, which is activated by neutrophil detection of a microbial threat *via* pattern recognition receptors and results in inflammatory cytokine production. This potent pro-inflammatory pathway is also the target of several immunosuppressive drugs used for the treatment of autoimmune disorders, during solid organ and hematopoietic cell transplantations, and as a part of anti-cancer therapy: but what effects these drugs have on neutrophil function, and their broader consequences for immune homeostasis and microbial defense are not yet known. Here, we bring together the emerging literature describing pathology- and drug- induced neutrophil impairment, with particular focus on their effects on calcineurin-NFAT signaling in the innate immune compartment.

## Introduction

Neutrophils are the most abundant cells of the innate immune system and play a key role in antimicrobial and antifungal defenses (1). At the site of infection, pathogens are detected *via* pattern recognition receptors (PRRs) whose ligation leads to the activation of a complex network of signaling cascades that together orchestrate the neutrophil’s ability to kill microbes *via* the generation of reactive oxygen species (ROS), degranulation of effector molecules, and the release of neutrophil extracellular traps (NETs) (2, 3). Moreover, these signaling pathways stimulate the production and release of chemokines and cytokines by neutrophils that act both locally, recruiting other immune cells to the site, and systemically, to regulate the wider anti-microbial immune response (4). An emerging player is the calcineurin (CN)- nuclear factor of activated T-cell (NFAT) pathway, which is activated by Ca^2+^ influx, leading to NFAT translocation to nucleus and modulation of gene transcription (5), and has critical roles both in myeloid cell function and immune homeostasis (6).

Neutrophils occupy a unique and critical functional niche within effective immunity, therefore it is perhaps unsurprising that impaired neutrophil function or regulation can have profound consequences: when neutrophils are unable to respond effectively this leads to increased susceptibility to infection, and negatively impacts the inflammatory and healing process (7–11), while their exaggerated activation can result in tissue damage (10, 12, 13). Neutrophil function may be impaired by another pathology, as in the case of sepsis (14), but the picture becomes even more complicated when the cause of neutrophil dysfunction is medication being used to treat an existing illness. Recent studies have shown that several types of therapy including corticosteroids (15), cytotoxic drugs (16), and cancer chemotherapy (17, 18) can affect neutrophil functions and that this process is linked with unfavorable treatment outcomes. The pro-inflammatory pathways operating in neutrophils may also be directly affected: calcineurin inhibitors (CNI) suppress activation of the CN-NFAT pathway and are now widely used in transplantation medicine to prevent graft versus host disease and also to treat autoimmune disorders as psoriasis, severe atopic dermatitis, and rheumatoid arthritis (19, 20). While these strategies may be effective in treating the primary condition, the downstream consequences of neutrophil impairment are only just starting to be understood. What appears to unify these diverse conditions is the emergence of neutrophil subsets with profoundly immunosuppressive properties, which are only just beginning to be understood. Here, we will review the latest findings on neutrophils and their functions during CNI-based immunosuppressive therapy and in the immunosuppressive milieu of sepsis, and ask what we can learn from these two situations that might advance our knowledge of neutrophil function/regulation and enable better clinical management of affected patients.

## Neutrophil Diversity in Health and Disease

For a long time, neutrophils were considered as a homogeneous population of innate myeloid cells, however, evidence has now accumulated that the population in fact exhibits considerable phenotypic and functional heterogeneity (21, 22), that is further expanded during certain pathologies or under specific conditions such as immunosuppression.

### Neutrophil Subsets: A Primer

Neutrophils produced in the bone marrow are dynamically released into circulation and within a few hours migrate to tissue (23). Despite the short life in circulation, neutrophils undergo morphological and phenotypical changes referred to as aging (24, 25). In this context, so-called fresh neutrophils are released from bone marrow, and they leave blood circulation as aged neutrophils (24). Dynamic of neutrophil release and clearance changes during day and levels of fresh and aged neutrophils follows natural circadian patterns (24). Several specific functional differences of the aged neutrophils have been described to date, including lower levels of L-selectin (CD62L), increased expression of β2-integrins, enhanced ROS production, and a higher tendency to undergo NETosis (25, 26). Moreover, aging primes neutrophils for a more aggressive inflammatory response *in vivo* (25, 26).

Normal density neutrophils (NDNs) traditionally separate in high density fraction after density gradient centrifugation. However, low density neutrophils (LDNs) are a suggested neutrophil subpopulation with a similar density to peripheral blood mononuclear cells (PBMCs) associated with a number of pathological conditions including systemic lupus erythematosus (27), sepsis (28), tuberculosis (29, 30), HIV (31), severe fever with thrombocytopenia syndrome (32), asthma (33), cancer (34–36), and it is associated with postoperative surgical stress in patients with gastrointestinal malignancies (37). Nevertheless, the impact of LDN in these pathologies has not been fully characterized. Recently, LDNs of healthy individuals show identical properties compared to NDNs (38). LDNs have a comparable ability of oxidative flare-ups, apoptosis, a similar effect on T-cell proliferation or IFN-γ production. Compared to NDNs, only a reduced ability of NETosis was found in LDNs in healthy donors (38). Interestingly, neutrophil stimulating agents (e.g. TNF, LPS, or fMLF) induced generation of LDNs from NDNs *in vitro* (38) suggesting that neutrophil density alone is insufficient to distinguish individual neutrophil subsets and functional or phenotypic differences need to be discovered.

While neutrophils have been considered as simply pathogen-killing cells for a long time, it is only more recently that their complex role in health and disease, in particular, in immunosuppression, have begun to be uncovered.

### Neutrophil Subsets Associated With Immunosuppression

The blood of healthy individuals contains both LDN and NDN (39). Recently, a population of “suppressive NDN” that is composed of mature activated cells have been defined (40). Further, in the condition of systemic inflammation, a subset of NDNs characterized by CD62^low^ CD11b^hi^ CD11c^hi^ that suppress T-cell proliferation through direct contact with the integrin Mac-1 was identified (41). Concurrently, recent studies revealed significant increases in the relative proportion of LDNs in patients with sepsis (28), infectious and autoimmune diseases (42), and cancer (34). Interestingly, a higher frequency of LDNs has also been found in immune-compromised patients, such as those infected with HIV or affected by common variable immunodeficiency (43). LDNs from these patients may also show profound functional alterations, for example in systemic lupus erythematosus where LDN exhibit enhanced spontaneous NETosis and relatively higher mitochondrial ROS production when compared to NDN *ex vivo* (44). In patients with cancer, circulating LDNs display marked immunosuppressive and pro-tumorigenic activities, similar to those documented for tumor-associated neutrophils (45–47).

These findings caused researchers to further dissect the LDN population, leading to the discovery of multiple neutrophil subsets that differ in morphology, abundance, and maturation and activation status depending on the underlying disease process (39, 48). However, the expanded LDN population in thrombocytopenia syndrome exhibiting severe fever (32), as well as in patients with malaria (49), or asthma (33) wasn´t found to be suppressive (48), and a recent study in patients with severe COVID-19 uncovered a population of CD16^int^ LDNs with a markedly inflammatory gene expression signature whose frequency correlated with disease severity (50). Thus, it appears that the heterogeneity within the population of LDN that is expanded in these disease states can accommodate the emergence of dominant immunosuppressive, non-suppressive and pro-inflammatory features. The question arises: are there any specific cell types within the LDN population that are strongly and specifically associated with immunosuppression?

Recent studies looking at immunosuppressive neutrophils have uncovered a potential candidate LDN subpopulation but have yet to agree on a unified terminology: these cells are variously termed “granulocytic-” or “polymorphonuclear- myeloid-derived suppressor cells” (G-MDSCs or PMN-MDSCs) (47, 48, 51). Although studies of these cells are in their infancy, we know that PMN-MDSCs often contain immature neutrophils (48, 52, 53) and are observed in patients with sepsis or fungal infections caused by *Aspergillus fumigatus (A. fumigatus)* or *Candida albicans* (*C. albicans)* (54, 55). *Ex vivo*, PMN-MDSCs from these patients inhibit T-cell proliferation and IFN-γ production *via* ROS or arginase-1 production (48, 54, 55). Identifying PMN-MDSCs among LDNs has been challenging as there is a lack of a defined, consensus surface molecule expression panel that can distinguish between the two (38, 47). However, it is possible to confirm MDSCs’ identity functionally, through their ability to suppress immune responses (e.g. inhibition of T cell proliferation or IFN-γ production), or by their expression of biochemical and molecular markers (such as the genes encoding ARG1, NOS2, NOX2, and TGF-β) (56). It is hoped that future studies, perhaps utilizing scRNA-seq, will be able to define a unique phenotype allowing reliable identification and isolation of these cells for further study: one of the most pressing questions is which molecular pathways are involved in normal neutrophil functions, and how are they altered in immunosuppressive neutrophil subsets.

## NFAT Signaling in Myeloid Immune Cells: Is This the Key Pathway Mediating Neutrophil Functions?

The CN-NFAT pathway, prototypically recognized in T cells (57, 58), has in recent years been identified as an important signaling cascade linking PRR activation, anti-microbial immunity, and myeloid cell functions (6, 59–61). Early work demonstrated NFAT activation in murine monocytes and DCs following the engagement of dectin-1 by zymosan or live *C. albicans* (62), with subsequent studies showing that this pathway can be stimulated by multiple pathogen-associated molecules, with varied outcomes. For example, bacterial LPS stimulation of murine DCs induced CD14-dependent extracellular Ca^2+^ influx, leading to CN-NFAT activation (63). Recently, these findings were enriched by a study that showed the importance of inositol triphosphate (IP_3_) receptor 3 (IP_3_R3) and IP3 kinase B (ITPKB) in LPS-induced NFAT activation in mouse and human DCs (64), signifying an atypical mechanism of Ca2^+^ mobilization leading to CN-NFAT activation in these cells.

CN-NFAT activation also occurs in human monocytes exposed to the ligand from *Saccharomyces cerevisiae (S. cerevisiae)* cell wall - zymosan (65), and similarly in murine macrophages and DCs after dectin-1 ligation by zymosan or live *C. albicans* (62). However, in macrophages exposed to *A. fumigatus* distinct pathway of TLR9- and phagocytosis-dependent NFAT activation mediated by Bruton’s tyrosine kinase (BTK), but independent on MyD88 has been reported (66).

Soon after the discovery that LPS stimulation induced CN-NFAT activation in murine DCs, similar findings were made in neutrophils, including induced expression of the genes involved in inflammation modulation including *IL-10, Cox2, Erg1*, and *Erg2* (67, 68). Furthermore, the engagement of dectin-1 by either zymosan or live *C. albicans* leading to CN-NFAT activation has also been confirmed in murine and human neutrophils (**Table 1**) (67, 68).

**Table 1:** PRR expressed by neutrophils and their signaling affected by calcineurin inhibitors demonstrated in neutrophils or derived from myeloid cells.

Ligand	PRR	Major signaling pathway	Inhibitor	TFs	Affected cell type/organ	Reference
PAM3CSK4	TLR2	MAPKs, NF-κB, PI3K-Akt, CN-NFAT	CsA	NFAT	Hu-mast cell	(69, 70)
LPS	CD14	CD14/ITPKB/IP_3_R3/Syk/PCγ2/IP_3_/Ca^2+^↑/CN-NFAT	FK506	NFAT	M-DC	(63, 64)
Unmethylated CpG motifs	TLR9	BTK/PCγ2/IP_3_/Ca^2+^↑/CN-NFAT	FK506	NFAT	M- MF, M- Neu	(66)
zymosan	Dectin-1	hemITAM/Syk/PCγ2/IP_3_/Ca^2+^↑/CN-NFAT	CsA, 11R-VIVIT	NFAT	M-MF, M-DC, M-Neu, Hu-Neu	(62, 68)
B2-integrins	Streptoccocal M1 protein complex with fibrinogen	Ca^2+^↑/CN/NFAT	A-285222	NFAT	M-lung, spleen, liver	(71)

M, murine; Hu, human; Neu, neutrophils; MF, macrophages; DC, dendritic cells; LPS, liposaccharide.

Taken together, these data indicate likely parallels between the pathways and effects of CN-NFAT activation in monocytes and neutrophils, further solidifying NFAT as a central player in myeloid cell immunity. However, while little direct research has been done into the role of CN-NFAT in driving neutrophil functions or in different neutrophil subsets, studies with patients undergoing immunosuppressive therapy with CNI, or with disease-associated immunosuppression, have generated intriguing insights into this topic.

## Impact of Immunosuppression on Neutrophil Function And its Clinical Consequences

Immunosuppression is a concerning state for the host, leaving it open to opportunistic infection with an unresponsive defense system. This situation can result directly from pathogen infection, but may also be therapeutically induced in the case of frank autoimmunity or the need for dampened reactivity following organ transplant. Here we will explore neutrophils’ involvement in two distinct but partially-overlapping scenarios: during CNI immunosuppressive therapy and in post-sepsis immunosuppression.

### Impact of Calcineurin Inhibitors on Neutrophils

Drugs inhibiting CN-NFAT signaling, such as tacrolimus (FK506) and cyclosporine A (CsA), are widely used to suppress T‐cell responses and prevent allograft rejection in transplantation medicine (20, 72). These clinically successful CNI were also recently repurposed to treat autoimmune disorders [reviewed in (59)] and as cancer chemotherapy considering that several cancer types have constitutively activated and overexpressed NFAT (73, 74). However, counterbalancing their potentially beneficial therapeutic role, a growing body of evidence indicates the potential impact of these compounds on myeloid cell function, leading to increased susceptibility to infections (59, 65, 66).

Different experimental models and clinical reports suggest that increased susceptibility to fungal and bacterial infections in patients treated with CNI is not a generic effect of inhibition of adaptive immune responses, but rather due to direct impairment of NFAT signaling in myeloid leukocytes, including neutrophils (**Table 2**) (6, 15, 59, 66, 68, 76, 78, 79). *In vitro*, treatment with CsA significantly inhibited the expression of NOD1, an intracellular receptor for bacterial peptidoglycan, in murine neutrophils, macrophages, and DCs (76). Similarly, *in vivo*, CsA-treatment of mice decreased the renal resistance to uropathogenic *Escherichia coli* infection associated with decreased expression of neutrophil-attracting chemokines CXCL2 and CXCL1, and myeloperoxidase (MPO) in mouse kidneys (76). Similarly, administration of CNI affected the production of CCL2, CCL7, CCL12, and delayed pathogen clearance in mice infected by *S. cerevisiae* (80). CsA treatment also negatively altered the phagocytic ability of neutrophils in the blood of human transplant recipients, which can be explained by the downregulation of Nod1 mRNA both *in vitro* and *in vivo* (76).

**Table 2:** Impact of impaired calcineurin-NFAT signaling on neutrophil functions during infection.

Pathogen	Experimental model/Patient’s cohort	Used inhibitor	Effect on neutrophil function	References
*Aspergillus fumigatus*	Patients after HSCT		Impaired *A.f.* hyphae growth inhibition	(15)
*Aspergillus fumigatus*	C57BL/6 WT mice and Rag2^-/-^ mice	FK506	Impaired neutrophil recruitment and fungal killing	(66)
*Aspergillus fumigatus*	zebrafish	FK506	Reduced neutrophil recruitment (and increased mortality)	(66)
*Aspergillus fumigatus*	Mice (BALB/c and C57BL/6)	FK506 with hydrocortisone	Unspecific effect leading to increased mice/host mortality	(75)
*Escherichia coli* (UPEC)	Mice	CsA	Impaired CXCL2 and CXCL1 production, decreased neutrophil recruitment/migration and phagocytic killing of UPEC	(76)
*Escherichia coli* (UPEC)	Renal transplant recipients	CsA	Defective NOD-1 mediated bacterial phagocytosis by neutrophils	(76)
*Candida albicans*	Mice (WT and Rag2^-/-^)	CsA	Decreased *ex vivo* killing of *C. albicans* by neutrophils leading to increased mortality	(68)
M1 protein from *Streptococcus pyogenes*	Mice	A-285222	Reduced neutrophil infiltration in lung	(77)

CsA, Cyclosporine A; FK506, Tacrolimus; UPEC, uropathogenic Escherichia coli.

These molecular effects of CNI upon neutrophils can have profound consequences, most clearly demonstrated in the case of infection with the normally-harmless opportunistic pathogen, *A. fumigatus*. Neutrophils are the main innate immune cell type interreacting with germinating *A. fumigatus* conidia and hyphae to prevent their growth (81, 82); however, the ability of neutrophils to interfere with *A. fumigatus* is significantly impaired in CNI-treated patients after hematopoietic stem cell transplant (15) – an effect that has been mechanistically linked with increased mortality in CNI-treated *A. fumigatus*-infected mice (83). The impact of impaired CN-NFAT signaling on patients’ susceptibility to *A. fumigatus* is even more marked during GvHD or solid organ transplantation (75), partially explaining the severe and frequently fatal complication of *A. fumigatus* infection in patients suffering from these conditions.

Such observations led to several studies *in vitro* and in animal models aiming to understand how CNI affected neutrophil responses to fungal infection. FK506 treatment of mice with pulmonary aspergillosis led to reduced cytokine/chemokine responses (TNF-α; IL-6; CXCL-1 and CCL-3), delayed neutrophil recruitment, decreased neutrophil influx, and reduced fungal killing in the lung (66). The lowered ability of CNI-exposed neutrophils to kill fungal pathogens seems also to be mechanistically linked to the lack of CN-NFAT activation: neutrophil degranulation is a Ca^2+^-dependent process (84), which is impaired in the presence of a range of different CNI (78, 85–87). Similarly, NETosis requires the mobilization of both extracellular and intracellular calcium pools and is reduced by treatment with CsA and the macrolide immunosuppressant ascomycin (88). Recent data have also indicated a link between CNI treatment and the development and accumulation of PMN-MDSCs (89), raising the possibility that CN-NFAT signaling is also required for normal neutrophil subpopulation homeostasis.

These findings demonstrate a novel, incompletely characterized, connection between CN-NFAT signaling in neutrophils and increased susceptibility to infections, operating both at the population (PMN-MDSC emergence) and individual cell level (inhibition of cytokine production, degranulation, and NETs generation). Moreover, such data also clearly illustrate the crucial role of CN-NFAT signaling in maintaining these processes in the disease-free host.

Given the profound effects of common immunosuppressive drugs targeting CN (CsA, FK506) on neutrophils, the potential benefits of their use need to be carefully considered in the context of the risk of increased susceptibility to serious opportunistic infections.

### Neutrophil Role in Sepsis-Induced Immunosuppression

Sepsis is a life-threatening condition caused by the dysregulated host response to infection and subsequent organ dysfunction (90). The host immune response during sepsis is complex, involving simultaneous activation of both excessive pro-inflammatory and anti-inflammatory processes (14), which ultimately results in disturbed homeostasis and profound post-sepsis immunosuppression: the predominant driving force for late morbidity and mortality in patients with sepsis (91, 92). Neutrophils are strongly affected by sepsis and are thought to play a critical role in determining the clinical outcome (93). Multiple neutrophil effector functions become impaired during the course of the disease (14, 94, 95), leading to functional paralysis of these cells in severe sepsis. One contributing factor is thought to be TLR-induced upregulation of G protein-coupled receptor kinase 2 (GRK2) expression, which ultimately desensitizes neutrophils to CXCL2 coming from the site of infection, thereby inhibiting several functions as well as their migration (93). Alongside, sepsis is associated with impaired neutrophil phagocytosis: in part, this can be linked with the release of immature neutrophils with limited phagocytic capacity from the bone marrow into the blood during sepsis and septic shock (96); but is also associated with decreased expression of the NOD1 receptor in patients with sepsis (97). These pathways are critically important, as impaired neutrophil phagocytic activity and reduced cell-surface CD64 (Fc gamma receptor) expression have been strongly correlated with poor outcomes in patients with sepsis (98). While there is some evidence from the CNI setting that NOD1-receptor-mediated neutrophil phagocytosis is CN-NFAT-dependent (76), this has yet to be assessed in cells from patients with sepsis.

In many of these studies, neutrophils are considered as a bulk population, but more recent work has begun to look at the possible involvement of the PMN-MDSCs in sepsis. Thought to arise as a result of the persistent immune activation and inflammatory environment, PMN-MDSCs have been detected in patients affected by sepsis (54, 99, 100) where they exhibit strong immunosuppressive activity (54, 101–103). A recent study showed that the frequency of PMN-MDSCs remain elevated also in sepsis survivors long after their recovery from sepsis, suggesting that these cells might contribute to the long-term adverse effects observed in sepsis survivors (100). These findings suggest a possible role of PMN-MDSCs in sepsis-induced immunosuppression and thus future research should be directed to elucidate their function and the underlying mechanisms involved.

## Future Perspectives

It is now clear that neutrophils play a decisive role in different pathologies and during immunosuppressive therapies. As effector cells of innate immunity, they are crucial for pathogen clearance, but they are also involved in host homeostasis: on the flip side, neutrophil dysregulation is implicated in the development/maintenance of the immunosuppressive microenvironment found in cancer and after sepsis, but also in the inflammatory response underlying autoimmunity. This dual role of neutrophils in opposing pathological processes reflects their plasticity and heterogeneity, as more recently evidenced by the identification of functionally and phenotypically distinct neutrophil subsets.

The specific activation or immunosuppressive status of neutrophils is tightly regulated through neutrophil signaling pathways: nevertheless, the details of these signaling pathways and specifically how they might be explored as potential targets of therapeutic interventions are not sufficiently described. One such knowledge gap that is beginning to be filled concerns the calcineurin/NFAT pathway which, although its presence and activity is sufficiently demonstrated in neutrophils, has yet to reveal the consequences of its signaling in these cells under different conditions. This is likely to prove key moving forward because a detailed understanding of the immunosuppressive context of downstream PRR signaling in neutrophils is needed in order to define the mechanistic aspects which may have potential medical implications. Some progress is already being made in this regard with the development of next-generation CNI, such as the *VIVIT* peptide, which selectively inhibits the calcineurin/NFAT interaction, but does not compromise calcineurin’s phosphatase activity (104). A recent study showed the potential of this peptide, conjugated to nanoparticles designed to target phagocytic cells, to dampen inflammation in a murine model of arthritis (64). Not only do such approaches hold direct therapeutic potential, but they may also prove invaluable as tools with which to discriminate the roles of CN-NFAT activation in specific cell types, including neutrophils.

Overall, there is emerging evidence pointing to the importance of the CN-NFAT axis in the myeloid cell/neutrophil response to pathogens and also the effects of its impairment on myeloid cell/neutrophil function during different disorders. Although CNI-mediated immunosuppression is applied in multiple situations, the broader adverse effect on the immune system needs to be carefully evaluated: the development of more specific therapeutic strategies targeting individual cell types or over-activated pathways should be considered a priority. Our knowledge of the biology of NFAT signaling in neutrophils is in its infancy and highlights the need for expanding our understanding of the molecular mechanisms modulating neutrophil functions in health and disease.

## Author Contributions

OV prepared the table, and wrote the manuscript. KB conceptualized, wrote and critically reviewed the manuscript. MZ, MH-K, and MV wrote and critically reviewed the manuscript. JF conceptualized, wrote and critically reviewed the manuscript. All authors contributed to the article and approved the submitted version.

## Funding

This study was supported by the European Social Fund and European Regional Development Fund—Proj- ect MAGNET (grant no. CZ.02.1.01/0.0/0.0/15_003/0000492) and ENOCH (CZ.02.1.01/0.0/0.0/16_019/0000868); and the Ministry of Health of the Czech Republic (grant nr. NU21-06-00408, all rights reserved) and DRO (Institute of Hematology and Blood Transfusion – UHKT, 00023736).

## Conflict of Interest

The authors declare that the research was conducted in the absence of any commercial or financial relationships that could be construed as a potential conflict of interest.

## Publisher’s Note

All claims expressed in this article are solely those of the authors and do not necessarily represent those of their affiliated organizations, or those of the publisher, the editors and the reviewers. Any product that may be evaluated in this article, or claim that may be made by its manufacturer, is not guaranteed or endorsed by the publisher.
